# Caddisfly (Trichoptera, Insecta) fauna and assemblages of the north-eastern part of the Pannonian Lowland (West Ukraine, Transcarpathia)

**DOI:** 10.3897/BDJ.10.e91004

**Published:** 2022-11-07

**Authors:** Kálmán Szanyi, Antal Nagy, Szabolcs Szanyi

**Affiliations:** 1 University of Debrecen, Juhász-Nagy Pál Doctoral School of Biology and Environmental Sciences, Debrecen, Hungary University of Debrecen, Juhász-Nagy Pál Doctoral School of Biology and Environmental Sciences Debrecen Hungary; 2 University of Debrecen, Faculty of Science and Technology, Department of Hydrobiology, Debrecen, Hungary University of Debrecen, Faculty of Science and Technology, Department of Hydrobiology Debrecen Hungary; 3 University of Debrecen Faculty of Agricultural and Food Sciences and Environmental Management, Institute of Plant Protection, Debrecen, Hungary University of Debrecen Faculty of Agricultural and Food Sciences and Environmental Management, Institute of Plant Protection Debrecen Hungary

**Keywords:** light trap, Bereg Plain, Caddisfly Conservation Index, mercury-vapour lamp, phenology, ecological preferences

## Abstract

The caddisfly fauna of the Transcarpathian part of the Pannonian Lowland was poorly studied formerly. Here, we present the results of a six-year survey (2015-2020) carried out in four sampling sites of the Ukrainian part of the Bereg Plain and provide the actualised checklist of this area. Actually, 7346 specimens of 53 caddisfly species were collected. The number of known caddisfly species increased from 13 to 61. Two species *Hydropsycheguttata* and *Parasetodesrespersellus*, which formerly were considered extinct in the Pannonian Ecoregion, were detected and another especially rare species (e.g. *Cyrnusflavidus)* was also recorded. The fauna of the region cover a significant part of both Hungarian and Ukrainian caddisfly fauna. Assemblages of four characteristic habitat types of the region showed significant differences considering their quantitative and qualitative composition, substrate, current, hydrological- and feeding types. The high diversity and natural value of the small lowland watercourses were proven using a new Caddisfly Conservation Index (CCI) calculated, based on vulnerability and rarity of species. The fauna and assemblages showed a unique character mainly independent from large rivers of the region.

## Introduction

Caddisflies are the most species-rich order of primarily aquatic insects having more species (more than 16,270 species) than all of the other orders (Holzenthal et al. 2007, Morse 2011, Adler and Foottit 2017, Morse 2017). They are considered one of the most useful and important aquatic organisms for monitoring water quality, as they are sensitive to high sediment and nutrient concentrations (Barbour et al. 1999, Jehamalar et al. 2010). Data on their occurrence and frequency are used in both biological status assessments and monitoring of water quality, many of which are now mandated by federal and municipal statutes in developed countries (Resh and Unzicker 1975, Resh and Rosenberg 1984, Lenat 1993, Resh 1993, Dohet 2002, Kiss 2002, Holzenthal et al. 2007, Graf et al. 2008, Jehamalar et al. 2010, Szanyi and Szanyi 2019). They are essential members of both aquatic and terrestrial food webs because of their amphibious habit (Morse 2017). The consumption of adults by terrestrial predators helps to ensure a return of nutrients from freshwater ecosystems to the surrounding terrestrial environment (Jackson and Resh 1989). In addition to this, various caddisfly larvae are able to consume organic matter that has inappropriate size for other groups of aquatic insects. Caddisfly larvae are able to engineer their habitats by linking mineral substrates with their silk, contributing to substrate stability (Cardinale et al. 2002, Statzner 2012, Albertson et al. 2014, Morse et al. 2019).

However, sampling caddisfly larvae and identifying them to species level can often be a difficult task. Thus, we can obtain a more accurate picture of the caddisfly fauna of a given area by examining their adults (Calor and Mariano 2012, Szanyi and Szanyi 2019). Adults are generally night-active, flying insects with positive phototaxis (Steinmann 1970); thus, one of the most effective methods for their sampling is light trapping (Széky 1977, Jermy 1998).

Lowlands have characteristic caddisfly fauna and assemblages because of the special character of watercourses with slow velocity, low oxygen concentration and heavy sediment loads (Skuja and Spungis 2010). The Pannonian Lowland has unique wildlife revealed in the case of many terrestrial (e.g. Lepidoptera (Szanyi et al. 2015a); Orthoptera (Szanyi et al. 2021); Coleoptera (Keszthelyi 2015); Aves (Ónodi et al. 2021); etc.) and aquatic taxa (e.g. Pisces (Antal et al. 2016); Amphibia (Vörös et al. 2016); etc.). The caddisfly fauna of the Pannonian Lowland is generally well-studied and discussed by Nógrádi and Uherkovich (2002); however, in thenorth-eastern part belonging to Ukraine, it is mainly unknown.

Investigation of the caddisfly fauna of Ukraine began in the 19^th^ century (Hagen 1858), but it is still relatively less well known due to the complex history of the country (Górecki 2011). The last checklist was published in 2008, which confirmed the occurrence of 218 species in Ukraine (Szczesny and Godunko 2008). This number has been increased to 223 by the work of Górecki (2011), Stibeltsov and Martynov (2012) and Stibeltsov (2013). The intensity of investigations shows large spatial differences (Czacharowski and Godunko 2006, Szczesny and Godunko 2008, Górecki 2011, Stibeltsov 2013). Caddisfly fauna of the mountainous areas (e.g. the Carpathians, Crimean Mountains) is more intensively studied, while caddisfly fauna of the lowland areas, especially in Transcarpathia, were sparsely studied. The latest checklist mentioned 11 species from this latter area, a total that does not even get close to the true species-richness. Most of the investigations there were made more than 50 years ago (Ivlev and Ivasik 1961). Further surveys were carried out in the area only in 2011, in which two new species were recorded (*Oecismusmonedula* and *Oecetistestacea*) (Górecki 2011).

Our first caddisfly samplings were made in the Transcarpathian part of the Bereg Plain between 2015 and 2016, providing the first data on the caddisfly fauna of the Velyka Dobron’ Game Reserve (Szanyi and Szanyi 2018, Szanyi and Szanyi 2019). The samplings were made with a Jermy-type light using a mercury-vapour lamp. Our preliminary data revealed a species-rich and vulnerable caddisfly fauna; thus, between 2015 and 2020, regular intensive studies were carried out in the area to collect and compare data on caddisfly assemblages living in the most characteristic habitat types of the region.

Here, we provide an actual checklist of the caddisfly fauna of the lowland part of Transcarpathia, based on published data and our intensive samplings and compare it with the fauna of the whole Pannonian Lowland. Assemblages of four characteristic habitat types are also compared considering their quantitative and qualitative composition and natural value.

## Material and methods

### Sampling sites

The collections were carried out in several areas of the Ukrainian part of the Bereg Plain. The examination of caddisflies must be made directly next to aquatic or wetland habitats; thus, the light traps were placed on different areas which are rich in these habitat types. Two sampling sites (VD1, VD2) were located within the area of the Velyka Dobron Game Reserve, in the vicinity of the Latorica River, the Szernye Marsh Canal and several smaller canals and wetlands. A third sampling site was in Tisauifalu (TF), next to the Csaronda and Tisza Rivers and another one between the villages of Bakosh and Demechi (BD), near to the Szernye Canal, Lónya Canal and several other small watercourses (Table 1).

The Bereg Plain is the part of the Upper Tisza region on the north-eastern part of the Pannonian Lowland. As this lowland area has significant Carpathian and continental climatic and biogeographic effects, it can be characterised by the richness of forests and wetlands (Szanyi et al. 2015b) and it has a cool (approximately 8.9°C) and relatively humid (mean precipitation: 609 mm/year) continental climate (Simon 1953).

### Data collection and samplings

The caddisfly fauna of the Transcarpathia was nearly unknown. In 2008, Szczesny and Godunko (2008) published a discussion on the caddisfly fauna of Ukraine in which only some scattered data could be found from Transcarpathia. After that, Górecki (2011), Stibeltsov and Martynov (2012) and Stibeltsov (2013) reported caddisfly data from Ukraine, but only Górecki (2011) provided data from Transcarpathia. These sources contain distribution data of only 13 species living in Transcarpathia.

During the study, a Jermy-type light trap with 125 W and then 80 W mercury-vapour lamp (HgLi) and portable light traps were used. In portable traps, three types of UV, a mixed-white fluorescence tube and two types of LED lamps were used (Table 1).

In the recent intensive period of samplings, a total of 114 samples were taken between 2015 and 2020 with a Jermy-type light trap which permanently worked in the VD1 site and with portable light traps in all the studied sites. In the VD1 site, samples were taken both with the Jermy-type light trap and portable light traps between 2015 and 2018. In the other three sites, portable light traps with different light sources were used in different years and combinations (Table 1).

Identification of the caught caddisfly specimens was made, based on the keys of Malicky (2004) and the nomenclature of Nógrádi and Uherkovich (2002) was followed. The genitalia of the female representatives of the Hydropsychidae species shows a high degree of similarity with each other. Thus, in the case of this family, reliable identification is only possible, based on the male individuals. So, in the present survey, only the Hydropsychidae males were identified at species-level.

### Data analysis

To characterise the fauna, the summarised checklist was made with published and our non-published distribution data. The composition of the fauna was compared with the fauna of the whole Pannonian Region and also with the fauna of Ukraine.

The sampling effort and the methods employed differed amongst the sampling sites. Therefore, when comparisons were made amongst assemblages at different sites, the common part of the datasets collected with the same methods was used.

During the comparison of habitat types, species-richness, quantitative composition of assemblages, substrate-, current- and hydrological preference and feeding type of species (Graf et al. 2008) were used.

To evaluate the diversity of the different sampling sites, Shannon-Wiener diversity indices were calculated. To evaluate the conservation value of the habitat types, a new caddisfly conservation index (CCI) was used, based on grasshopper conservation indices published by Matenaar et al. (2015) and Szanyi et al. (2021). CCI was calculated, based on local rarity and vulnerability of species. Both parameters were summed for each species and divided by eight (the maximum value) to obtain a CCI value between zero and one. In the case of dispersal capacity and rarity, the original method was followed, but the parameters were grouped in four categories. The local rarity was measured upon the relative frequencies (RF%) of species in the studied four sites. A species was considered as common (= 1; RF% > 0.01530), frequent (= 2; RF% = 0.00345-0.01530), low frequency (= 3; RF% = 0.00108-0.00345) and rare (= 4; RF% < 0.00108). In the case of vulnerability, categories not threatened (= 1), threatened (= 2), vulnerable (= 3) and endangered (= 4) species were used. Since the categorisation of caddisfly species, based on conservational status in Ukraine, has not been made until now and the studied area is a part of the Pannonian Lowland, categories of Nógrádi and Uherkovich (2002), established for the Hungarian fauna, were used. The two parameters were summed and divided by eight (the maximum value) to obtain a CCI value between zero and one. The CCI values of the study sites were determined as a sum of the values of the species of the given site. The standardised caddisfly conservation index (CCI) was also calculated for sites by dividing CCI by the number of species on the given site. While the CCI values depend on both species number and the value of the species, the CCIs are not influenced by the species-richness (Matenaar et al. 2015, Szanyi et al. 2021).

## Results

### Fauna

Before 2015, only 13 caddisfly species were reported from Transcarpathia (Table 2). During our 6-year study, 7346 specimens of 53 caddisfly species were collected from four sampling sites. The actual checklist of the region contains 61 species which is 29.05% of the Pannonian (210 species; Nógrádi and Uherkovich 2002) and 27.98% of the Ukrainian fauna (218 species; Szczesny and Godunko 2008, Górecki 2011, Stibeltsov and Martynov 2012, Stibeltsov 2013). *Hydropsycheguttata, Ceracleariparia, Oecetistestacea, O. tripunctata* and *Parasetodesrespersellus* are rare, while *Oecetistestacea* has long been unknown in Ukraine. Górecki (2011) provided the first and the only data of this species from Transcarpathia. *Parasetodesrespersellus*, *Ceracleariparia* and *Oecetistripunctata* were first found in south-eastern Ukraine by Stibeltsov and Martynov (2012) and by Stibeltsov (2013) and Buczynska et al. (2014). There are numerous data for *Hydropsycheguttata*; however, these data were found to be incorrect identifications, based on Czacharowski and Godunko (2006) and Szczesny and Godunko (2008). Regarding the Hungarian fauna, *Hydropsycheguttata* and *Parasetodesrespersellus* are extinct in Hungary and only one individual of *Cyrnusflavidus* has been caught from the whole ecoregion, until now (Nógrádi and Uherkovich 2002).

Regarding the classification of Nógrádi and Uherkovich (2002), beyond the two mentioned extinct species, two directly endangered (*Polycentropusirroratus, Oecetistestacea*), seven endangered (*Cheumatopsychelepida, Trichostegiaminor, Limnephilusxanthodes, Silopallipes, Ceracleariparia, Mystacidesazureus* and *Oecetistripunctata*) and 16 vulnerable species were recorded.

Eight of the formerly-known species (*Rhyacophilanubila*, *Ptilocolepusgranulatus*, *Hydropsycheincognita*, *Polycentropusflavomaculaus*, *Brachycentrussubnubilus*, *Grammotauliusnitidus*, *Athripsodesaterrimus* and *Oecismusmonedula*) were not caught during the present intensive studies.

Captured species were distributed in 28 genera of 10 families. The most species (47) were caught in the VD1 site in Velyka Dobron’, where the sampling effort was higher than in the other three sites. In the VD2 site, 17 species, in the BD site 27 and in the TF site 21 species were identified (Table 2). Most species belong to the families Leptoceridae (17) and Limnephilidae (15), while the most numerically abundant families were the Hydropsychidae (with 2717 individuals) and the Leptoceridae (with 2396 individuals). The five most abundant species were *Leptocerustineiformis* (1244), *Ecnomustenellus* (678), *Limnephilusflavicornis* (613), *Ceracleadissimilis* (478) and *Oecetisnotata* (395). The species *Leptocerustineiformis* represented 16.7% of the total specimens collected.

### Assemblages of sampled habitats

The dataset used for comparison of the four sampling sites consisted of 2321 specimens distributed amongst 35 species. The sampling site near Bakosh Village (BD) was the most species-rich (with 27 species), while caddisfly assemblages of the TF site were the most abundant. The VD2 site near Velyka Dobron’ had only 13 species with especially low abundances (Table 3).

Regarding the caddisfly conservation index, the most valuable assemblage lived in the most species-rich site BD and the value of the other sites correlate with the value of species-richness. The values of the standardised CCIs index were nearly equal in different sites (Table 3). The Shannon-Wiener diversity values did not correlate with the value of species-richness. The highest value belonged to the BD site, but it was followed by the VD1.

Regarding the diversity of different categories of the studied traits of caddisfly assemblages, nearly all of them appeared in all studied sites, but their relative frequencies showed remarkable differences.

The relative frequencies of the species belonging to different substrate types showed remarkable differences. The species assemblage of the VD1 site was dominated by species living on particulate organic matter (POM), in the VD2 site eurytopic species were extremely dominant and in the other two sites, species which utilised macrophytes as a substrate showed the largest relative frequencies. In the latter two sites, the eurytopic species also reached relatively high frequencies, contrary to the VD1 site where their ratio was only 10.19% (Table 3).

Regarding current types, the VD1 site showed a unique character with high relative frequencies of limnophil (50.32%), rheobiont (21.6%) and limnobiont (20.99%) species. The assemblages living in the other three sites were dominated by limnobiont species, especially in the case of the VD2 site, while the frequencies of other current types were under 15.00% (Table 3).

In the VD1 site, species belonging to the eurytopic hydrological type were dominant (54.32%), followed by the frequencies of eupotamon type species (22.53%). The assemblage of the VD2 site was characterised by paleo- and plesiopotamon type species (82.20%). The other two assemblages (in BD and TF sites) were similar in character, with the dominance of paleopotamon type species followed by the nearly equal values of eupotamon and paleo- and plesiopotamon types (Table 3).

Considering the frequencies of feeding types, the pattern was the same as that found for hydrological types. The assemblages living in BD and TF sites showed large similarities with high frequencies of “grazers and scrapers and shredders” and predator categories. In the quite different VD2 site, predators showed extreme dominance with a relative frequency of 83.77%. The VD1 site also has a unique character with a high frequency of “predators and shredders” (59.88%) type species followed by “passive filter feeders and predators” (17.28%) (Table 3).

## Discussion

The number of known caddisfly species of Transcarpathia (west Ukraine) increased from 13 to 61 during our 6-year study. Eight of the 13 formerly-recorded species have not been found in recent surveys (Szczesny and Godunko 2008). Most of them prefer watercourses of hilly and mountainous areas with moderate or fast water velocity, which may explain their absence (Nógrádi and Uherkovich 2002). Based on this information, the caddisfly fauna of the Transcarpathian Lowland was formerly virtually unknown. This newly-defined fauna represents a significant part of the known Ukrainian (27.98%) and even the Hungarian (29.05%) fauna, which include the Pannonian Lowland (Nógrádi and Uherkovich 2002, Szczesny and Godunko 2008, Górecki 2011, Stibeltsov and Martynov 2012, Stibeltsov 2013).

*Hydropsycheguttata* and *Parasetodesrespersellus* are considered extinct from the area of the Pannonian Lowland (Nógrádi and Uherkovich 2002). The former species was rare originally, while the other had stable populations until the 1980s (Újhelyi 1971). Since then, adults of neither species have been found, despite numerous samplings. According to Nógrádi and Uherkovich (2002), the distribution of *P.respersellus* is now restricted to the eastern part of its former distribution; this accords with our sampling, in which the species was collected in the eastern part of the Pannonian Lowland.

The BD site was the most species-rich, contrary to its low habitat diversity, with only small canals and channels. The high number of *Leptocerustineiformis* specimens (739) accounts for the higher abundance and relatively low Shannon-Wiener diversity value in the TF site. The VD1 and VD2 sites were close to each other and both of them had almost the same kind of waterbodies. Despite this, their caddisfly fauna showed large differences. The least number of species and individuals were caught in the VD2 site. There, traps were located next to a fishpond lake, in which only the most common and eurytopic species occurred. This is also reflected by the low CCI number, which considers the sensitivity of the caught species. A very common and widespread species, *Ecnomustenellus*, was highly dominant in this site, accounting for the low Shannon-Wiener diversity value.

The particulate organic matter (POM) related species were dominant in the VD1 site, based on the substrate preferences, while the species which need presence of the macrophytes were characteristic to the BD and TF sites. This can be attributed to the Szenye Marsh channel next to the VD1 site having a eutrophic character and rich in organic matter, while the waterbodies near the BD and TF sites had dense macro vegetation coverage. The VD2 site is also strongly characterised by eurytopic species for the substrates.

In the case of the waterbodies near to the VD2 and BD sites, there was no significant water velocity, thus its fauna can be described with limnobiont species. The high abundance of the limnobiont/limnophil species is also characteristic to the VD1 and TF sites. The rheobiont species also occurred in the traps of the VD1 site because of the proximity of the Latorica River. Although the TF site was close to the Tisza River, the effect of the River on the fauna could not be proved since there were no rheobiont species in the samples. This pattern can also be observed in the hydrological preferences of the species.

As the BD and TF sites were rich in macro vegetation, their fauna was dominated by species belonging to the grazer, scraper and shredder feeding types. In the VD1 site, besides the predators and shredders, the ratio of the passive filter feeders was also high. This feeding type needs a certain water velocity, which is provided by the Latorca River nearby. As the lake next to the VD2 site is poor in available nutrients, it is characterised mainly by predators.

The caddisfly fauna of the Transcarpathian part of the Pannon Ecoregion was mainly unknown (Szczesny and Godunko 2008) since previous researchers focused mainly on the caddisflies of mountainous areas (Górecki 2011). Here, we proved that the small lowland watercourses of this area have very unique and valuable caddisfly assemblages. Several rare and endangered species were found there and some of them were formerly considered extinct from the Pannonian Lowland. Most species detected during the investigation came from these small watercourses, while the Tisza or Latorica Rivers had a smaller effect on the composition of the fauna than expected. The Transcarpathian Lowland is rich in semi-natural sites of these kinds of wetlands endangered by natural and anthropogenic drought and high value of pollution (e.g. illegal deposition of communal waste and plastic pollution). The conservation of their high natural value showed by uniqe and diverse caddifly assemblages needs active intervention of both government policies and non-goverment organisations.

## Figures and Tables

**Table 1.** Characteristics of sampling sites studied between 2015 and 2020 with data of sampling methods (years/number of samples).

	**VD1**	**VD2**	**BD**	**TF**
township	Velyka Dobron’	Velyka Dobron’	Bakosh-Demechi	Tisauifalu
GPS (N/E)	48.444°N, 22,407°E	48.451°N, 22.392°E	48.407°N, 22.346°E	48.411°N, 22.267°E
Habitat types	medium and small-sized watercourses with slow water current	small canals, wetlands and a fishpond lake	only small canals and channels	a river and a small canal
**Traps**				
Jermy-type with mercury-vapour lamp	2015-2018 (83)			
Portable trap: White	2018 (14)	2019 (7)	2020 (6)	2020 (6)
Portable trap: UV1	2018 (14)	2019 (7)		
Portable trap: UV2	2018 (14)	2019 (7)	2020 (6)	2020 (6)
Portable trap: UV3	2018 (14)	2019 (7)		
Portable trap: LED1	2018 (14)	2019 (7)	2020 (6)	2020 (6)
Portable trap: LED2	2018 (14)	2019 (7)		

**Table 2.** Checklist of the Caddisfly fauna of the Ukrainian part of the Bereg Plain (Transcarpathia, W Ukraine). For details of sampling sites (VD1-TF), see Table 1.

**Family**	**Species**	**Publ.**	**VD1**	**VD2**	**BD**	**TF**
Rhyacophilidae	*Rhyacophilanubila* (Zetterstedt, 1840)	+				
Glossomatidae	*Agapetuslaniger* Pictet, 1834				+	+
Ptilocolepidae	*Ptilocolepusgranulatus* (Pictet, 1834)	+				
Hydroptilidae	*Agrayleasexmaculata* Curtis, 1834				+	
Hydropsychidae	*Cheumatopsychelepida* (Pictet, 1834)		+			+
Hydropsychidae	*Hydropsychebulbifera* McLachlan, 1878	+	+		+	
Hydropsychidae	*Hydropsychebulgaromanorum* Malicky, 1977		+	+	+	+
Hydropsychidae	*Hydropsychecontubernalis* McLachlan, 1865	+	+	+	+	+
Hydropsychidae	*Hydropsycheguttata* Pictet, 1834		+			
Hydropsychidae	*Hydropsycheincognita* (Pitsch, 1993)	+				
Hydropsychidae	*Hydropsychemodesta* Navàs, 1925	+	+	+	+	+
Hydropsychidae	*Hydropsycheornatula* McLachlan, 1878		+		+	+
Hydropsychidae	*Hydropsychepellucidula* (Curtis, 1834)		+	+	+	
Polycentropodidae	*Cyrnuscrenaticornis* (Kolenati, 1859)				+	+
Polycentropodidae	*Cyrnusflavidus* McLachlan, 1864		+			
Polycentropodidae	*Holocentropuspicicornis* (Stephens, 1836)		+		+	
Polycentropodidae	*Neureclipsisbimaculata* (Linnaeus, 1758)		+	+	+	+
Polycentropodidae	*Polycentropusflavomaculaus* (Pictet, 1834)	+				
Polycentropodidae	*Polycentropusirroratus* Curtis, 1835			+		
Psychomyidae	*Psychomyiapusilla* (Fabricius, 1781)	+	+	+	+	+
Psychomyidae	*Lypephaeopa* (Stephens, 1836)		+	+		+
Ecnomidae	*Ecnomustenellus* (Rambur, 1842)		+	+	+	+
Phryganeidae	*Agrypniavaria* (Fabricius, 1793)		+	+	+	
Phryganeidae	*Trichostegiaminor* (Curtis, 1834)		+	+		
Brachycentridae	*Brachycentrussubnubilus* (Curtis, 1834)	+				
Limnephilidae	*Limnephilusaffinis* Curtis, 1834		+			
Limnephilidae	*Limnephilusauricula* Curtis, 1834		+			
Limnephilidae	*Limnephilusdecipiens* (Kolenati, 1848)		+			
Limnephilidae	*Limnephilusflavicornis* (Fabricius, 1787)		+			
Limnephilidae	*Limnephilushirsutus* (Pictet, 1834)		+			
Limnephilidae	*Limnephilusincisus* Curtis, 1834		+			
Limnephilidae	*Limnephiluslunatus* Curtis, 1834		+			
Limnephilidae	*Limnephilusrhombicus* (Linnaeus, 1758)		+			
Limnephilidae	*Limnephilusxanthodes* Mclachlan 1873		+			
Limnephilidae	*Limnephilusvittatus* (Fabricius, 1798)		+			
Limnephilidae	*Glyphotaeliuspellucidus* (Retzius, 1783)		+	+	+	
Limnephilidae	*Grammotauliusnigropunctatus* (Retzius, 1783)		+			
Limnephilidae	*Grammotauliusnitidus* (Müller, 1764)	+				
Limnephilidae	*Halesustessellatus* (Rambur, 1842)		+			
Limnephilidae	*Micropternatestacea* (Gmelin, 1789)		+			
Limnephilidae	*Stenophylaxpermistus* McLachlan, 1895		+			
Goeridae	*Silopallipes* (Fabricius, 1781)				+	
Leptoceridae	*Athripsodesaterrimus* (Stephens, 1836)	+				
Leptoceridae	*Athripsodescinereus* (Curtis, 1834)		+	+	+	+
Leptoceridae	*Ceracleadissimilis* (Stephens, 1836)		+	+	+	+
Leptoceridae	*Ceracleariparia* (Albarda, 1874)		+		+	+
Leptoceridae	*Ceracleasenilis* (Burmeister, 1839)		+			
Leptoceridae	*Leptocerustineiformis* Curtis, 1834		+		+	+
Leptoceridae	*Mystacidesazureus* (Linnaeus, 1761)		+		+	
Leptoceridae	*Mystacideslongicornis* (Linnaeus, 1758)		+			
Leptoceridae	*Mystacidesniger* (Linnaeus, 1758)		+			+
Leptoceridae	*Oecetisfurva* (Rambur, 1842)		+		+	
Leptoceridae	*Oecetislacustris* (Pictet, 1834)		+		+	+
Leptoceridae	*Oecetisnotata* (Rambur, 1842)		+	+	+	+
Leptoceridae	*Oecetisochracea* (Curtis, 1825)					+
Leptoceridae	*Oecetistestacea* (Curtis, 1834)	+	+	+		
Leptoceridae	*Oecetistripunctata* (Fabricius, 1793)		+		+	+
Leptoceridae	*Parasetodesrespersellus* (Rambur, 1841)		+			
Leptoceridae	*Triaenodesbicolor* (Curtis,1834)		+	+	+	
Leptoceridae	*Setodespunctatus* (Fabricius, 1793)		+		+	+
Sericostomatidae	*Oecismusmonedula* (Hagen, 1859)	+				
**Total number of species**		**13**	**47**	**17**	**27**	**21**

**Table 3.** Number of caught species and individuals, values of Shannon-Wiener diversity index, values of caddisfly conservation indices (CCI and CCIs) and relative frequencies (%) of species belonging to different substrate, current, hydrological and feeding types in the studied sampling sites of Transcarpathian Lowland. For details of sampling sites (VD1-TF), see Table 1.

	**VD1**	**VD2**	**BD**	**TF**	**SUM**
Number of species	19	13	27	21	35
Number of individuals	324	191	464	1342	2321
Shannon-Wiener diversity	1.801	0.871	2.128	1.495	2,065
CCI	8.625	5.75	13.5	10.125	18.5
CCIs	0.45	0.44	0.50	0.48	0.53
**Substrate**					
eurytopic	10.19	84.29	27.16	32.12	32.36
algae	0.00	0.00	0.22	0.00	0.04
micro- and macrolithal	22.53	12.57	19.40	11.55	14.74
macrophytes	0.31	0.00	42.46	55.07	40.37
macrophytes and woody debris	0.31	0.00	0.22	0.07	0.13
macrophytes and pelal	0.00	0.00	0.65	0.15	0.22
macrophytes and POM	12.35	2.09	4.74	0.07	2.89
POM (particulate organic matter)	50.00	0.00	0.22	0.00	7.02
POM and woody debris	3.09	0.52	1.72	0.00	0.82
psammal and akal	0.31	0.00	3.23	0.89	1.21
woody debris	0.93	0.52	0.00	0.07	0.22
**Current type**					
limnobiont	20.99	84.29	63.36	74.07	65.36
limnophil	54.32	0.00	0.43	0.07	7.71
limno- and rheophil	0.93	3.14	14.44	3.95	5.56
rheophil	1.23	1.05	8.41	14.75	10.47
rheobiont	21.60	10.99	13.36	7.08	10.69
indifferrent	0.93	0.52	0.00	0.07	0.22
**Hydrological type**					
eurytopic	54.32	0.00	0.43	0.07	7.71
eupotamon	22.53	12.04	19.61	21.31	20.38
eu- and parapotamon	2.16	3.14	4.96	1.34	2.33
parapotamon	0.00	0.52	11.85	3.28	4.31
paleopotamon	0.31	0.00	42.89	55.22	40.54
paleo- and plesiopotamon	9.88	82.20	20.26	18.78	23.05
paleop and temporary water bodies	2.47	0.00	0.00	0.00	0.34
temporary water bodies	8.33	2.09	0.00	0.00	1.34
**Feeding type**					
eurytopic	0.31	0.00	4.09	1.19	1.55
gatherers/collectors	0.31	0.00	0.22	0.07	0.13
grazers and scrapers	1.23	1.57	4.53	4.40	3.75
grazers and scrapers and shredders	0.00	0.00	42.03	55.07	40.24
passive filter feeders	4.32	5.24	1.72	0.60	1.72
passive filter feeders and predators	17.28	5.76	11.64	6.48	8.96
predators	7.41	83.77	31.03	22.28	27.01
predators and grazers and scrapers	8.33	2.09	0.00	0.00	1.34
predators and shredders	59.88	0.52	3.45	0.07	9.13
shredders and gatherers/collectors	0.93	1.05	1.08	9.84	6.12
other feeding types	0.00	0.00	0.22	0.00	0.04
